# Physical controls and ENSO event influence on weathering in the Panama Canal Watershed

**DOI:** 10.1038/s41598-020-67797-7

**Published:** 2020-07-02

**Authors:** Devin F. Smith, Steven T. Goldsmith, Brendan A. Harmon, Jorge A. Espinosa, Russell S. Harmon

**Affiliations:** 1 School of Earth Sciences, Ohio State University, Columbus, OH 43210 USA; 2 Department of Geography and the Environment, Villanova University, Villanova, PA 19085 USA; 3 School of Landscape Architecture, Louisiana State University, Baton Rouge, LA 70803 USA; 4 Panama Canal Authority (Retired), Panama, Republic of Panama; 5 Department of Marine, Earth Atmospheric Sciences, North Carolina State University, Raleigh, NC 27695 USA

**Keywords:** Element cycles, Environmental sciences

## Abstract

Recent empirical studies have documented the importance of tropical mountainous rivers on global silicate weathering and suspended sediment transport. Such field studies are typically based on limited temporal data, leaving uncertainty in the strength of observed relationships with controlling parameters over the long term. A deficiency of long-term data also prevents determination of the impact that multi-year or decadal climate patterns, such as the El Niño Southern Oscillation (ENSO), might have on weathering fluxes. Here we analyze an 18-year hydrochemical dataset for eight sub-basins of the Panama Canal Watershed of high-temporal frequency collected between 1998 and 2015 to address these knowledge gaps. We identified a strongly positive covariance of both cation (Ca^2+^, Mg^2+^, K^+^, Na^+^) and suspended sediment yields with precipitation and extent of forest cover, whereas we observed negative relationships with temperature and mosaic landcover. We also confirmed a statistical relationship between seasonality, ENSO, and river discharge, with significantly higher values occurring during La Niña events. These findings emphasize the importance that long-term datasets have on identifying short-term influences on chemical and physical weathering rates, especially, in ENSO-influenced regions.

## Introduction

Empirical studies over the past two decades have documented the importance of small mountainous rivers (SMRs), particularly those in the tropics, on global silicate weathering and associated CO_2_ consumption budgets. In a search for controls, studies have recognized the strong positive feedback between physical and chemical weathering^1–3^ and noted the importance of underlying volcanic lithologies on maintaining elevated chemical yields^4–11^. Others have identified strong correlations/associations between weathering yields and precipitation/runoff^2,3,9,10,12^ and, more recently, land use/landcover practices^10,11,13^. Yet, despite their important contribution to silicate weathering, few datasets for SMRs exist at high temporal resolution^10,14^ and/or duration^15^ for such high-yield terrains. This lack of long-term records of high temporal resolution requires caution in generalizing the aforementioned statistical relationships, as years of anomalously high or low precipitation can result in misinterpretation of silicate weathering rates. This is particularly important as SMRs are presently being incorporated into newer global chemical weathering and associated CO_2_ drawdown models^16–18^. Furthermore, the absence of reliable long-term datasets prevents insight into the impact that multi-year or decadal climate patterns, such as the Pacific Decadal Oscillation (PDO) and El Niño Southern Oscillation (ENSO), can have on weathering fluxes.

In the tropics of the Caribbean and South America, ENSO events have been shown to have a pronounced and varied effect on rainfall^19–22^, streamflow^22–24^, soil moisture^23^, and vegetation^23,25^. For example, a review of hydrological data for Colombia observed that ENSO effects between 1997 and 1999 were stronger for stream flow than for precipitation, due to concomitant effects on soil moisture and evapotranspiration, with lower than normal soil moisture and stream flow occurring during El Niño conditions and the reverse situation pertaining during La Niña episodes^23^. Similar trends with ENSO events have been observed in Panama. For example, a previous study documented that an average of 8% less rainfall was received across almost all regions of Panama during 13 El Niño episodes between from 1920 to 1983^19^. Such ENSO-related reductions in precipitation and consequent runoff^26–28^ cause water supply issues for operation of the Panama Canal, which requires nearly 200,000 m^3^ of water per vessel transit^29^. It follows that these pronounced hydrological anomalies should also impact weathering fluxes, but this has not been documented to date.

**Figure 1:**
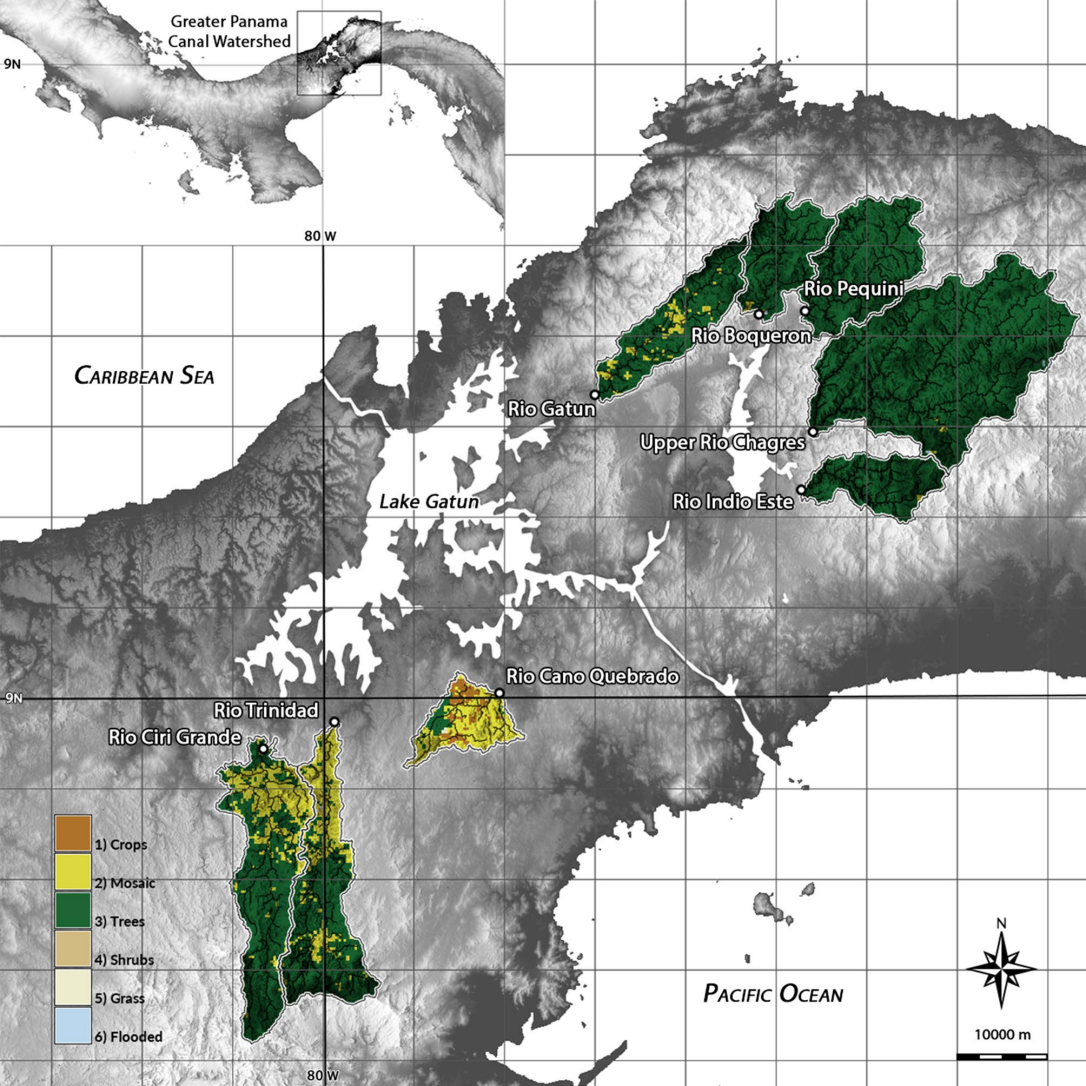
View of the eight sub-watersheds of the greater Panama Canal Watershed (PCW) included in this study. Landcover data is sourced from the European Space Agency (ESA) Climate Change Initiative (CCI) Landcover Dataset. Map generated in GRASS GIS 7.4 (GRASS Development Team, 2018; https://grass.osgeo.org/) by the authors.

The Panama Canal Watershed (PCW; 2,982 km^2^; 9° N, 80° W), comprised predominantly of eight major sub-watersheds, offers an ideal location to evaluate the physical and climatic controls on long-term weathering rates and CO_2_ consumption in tropical SMRs (Fig. 1). For example, the largely forested, steeply-sloping sub-watersheds on the north side of the canal—Río Gatún, Río Boquerón, Río Pequení, Río Chagres, and Río Indio Este, differ markedly from their mostly deforested, gently-sloping counterparts to the south—Río Cano Quebrado, Río Trinidad, and Río Cirí Grande^30,31^. The region also exhibits a strong trans-isthmus rainfall gradient, with the amount of precipitation received on the windward Atlantic coast more than twice that received on the leeward Pacific coast (~ 4,000 mm/year versus < 1,800 mm/year)^32^. Lastly, pronounced differences in regional precipitation values during ENSO events permit an evaluation of the impact of short-term climate patterns on weathering fluxes^21,26^. Here we utilize a robust, high-temporal frequency, hydrochemical dataset collected over 18 years between 1998 and 2015 by the Panama Canal Authority (ACP) to calculate annual and long-term cation and sediment fluxes for the PCW. We compare the resulting fluxes to potential controlling variables, such as mean annual rainfall and temperature, stream gradient and land use/landcover, both to identify compositional controls on river chemistry and to determine the strength and variability of controlling relationships over annual and decadal time scales. We also used this dataset to explore the statistical relationship between weathering fluxes and ENSO events.

### Climatic, geologic, and geomorphologic influences on weathering rates

**Figure 2:**
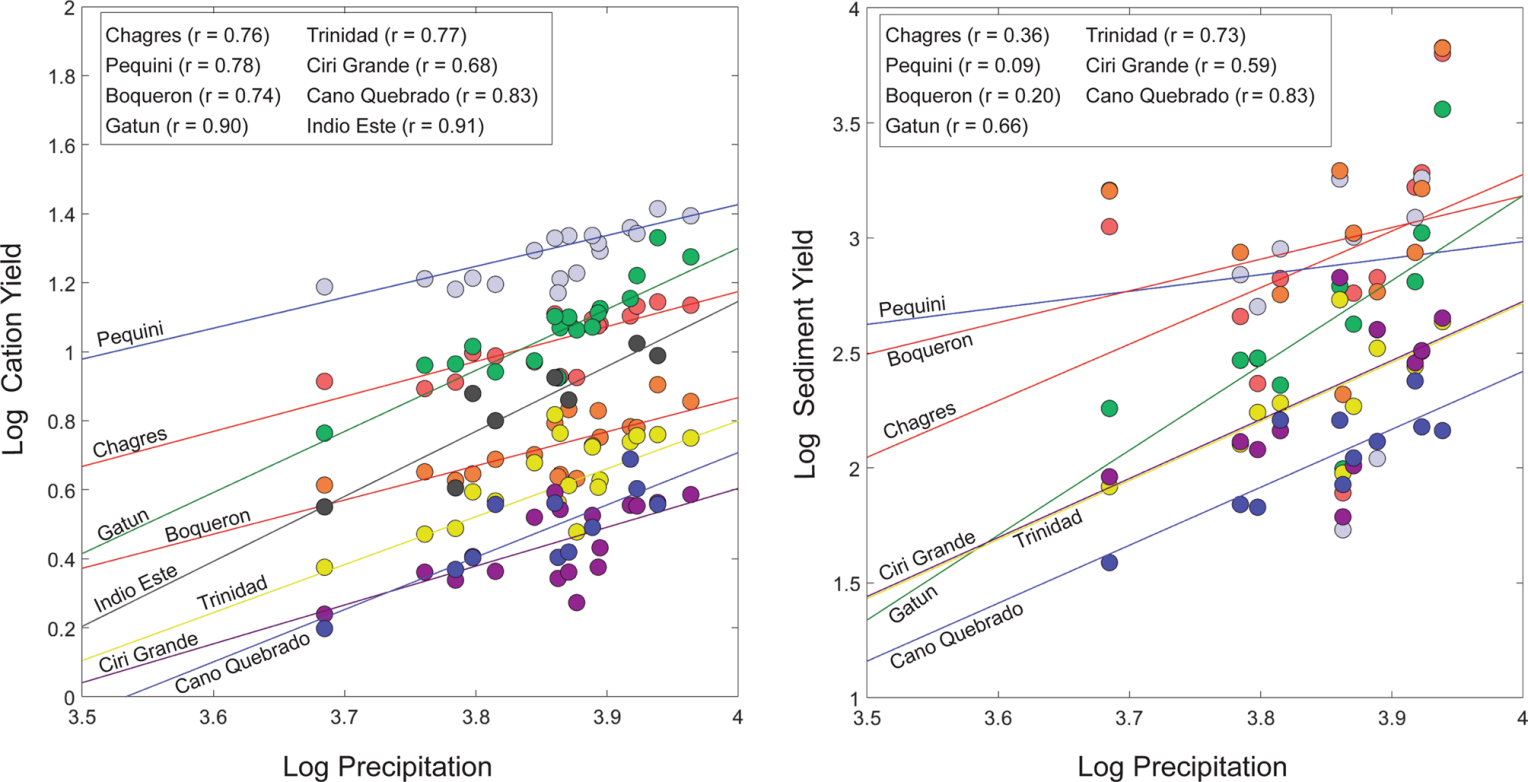
Effect of annual average precipitation on cation and sediment yields for each of the study watersheds. Removal of the two outliers for the comparison between sediment load and precipitation for the Pequini watershed improves the r value from 0.09 to 0.48.

Our long-term cation yields (corrected for sea salt contribution in precipitation and non-silicate contribution of Ca and Mg) for the PCW range from 2.85 to 19.3 t/km^2^/year, whereas our suspended sediment yields range from 124 to 1,494 t/km^2^/year (Table 1; Supplementary Tables 1 and 2). Our cation yields are in the lower range of those previously determined for watersheds across the Panamanian isthmus^10^, whereas our suspended sediment yields are within the range of those calculated using an earlier dataset for the PCW^31^. When we analyzed each river individually over the length of the study period using a Pearson correlation, all but one watershed showed a statistically significant (*p* < 0.05) positive relationship (*r*_avg_ ≥ 0.75) between cation yields and mean annual precipitation at Lake Gatun (Fig. 2; Supplementary Table 3). We observed a similar, but slightly more variable relationship (*r*_avg_ = 0.50) between sediment yields and mean annual rainfall (Supplementary Table 4). Although we identified no discernable pattern between correlation strength and geographic location, we did observe regional differences in weathering rates. For example, a two-tailed t-test revealed significant differences in both cation (α = 0.05, *p* = 0.038) and suspended sediment (α = 0.05, *p* = 0.011) yields between the north and south watersheds despite no noted differences in average discharge values (α = 0.05, *p* = 0.28). Our observed lack of statistically significant differences in discharge points to the importance of lithology, as well as other variables, in maintaining high chemical erosion rates. For example, watersheds on the north side of the canal are largely underlain by mafic to intermediate volcanic rocks compared to the largely sedimentary cover for those on the south side^11^. The importance of volcanic lithology in maintaining disproportionately high cation yields was previously documented as part of an isthmus-wide study^10,11^. While we observed a negative, and somewhat variable statistically significant relationship between cation yields and basin-wide mean annual temperature (*r*_avg_ = − 0.39), this is likely due to increased cloud cover associated with precipitation events. This idea is supported by an observed negative statistical relationship between basin wide mean annual rainfall and temperature (*r* = − 0.47, *p* < 0.05) over the study period.

**Table 1:** Long-term average cation and suspended sediment fluxes and other key environmental properties for the PCW.

River	Area upstream of sampling location (km^2^ )	Mean elevation (m)	Mean slope	Long-term average cation weathering flux (t km^–2^ year^–1^)^a^	Long-term average suspended sediment flux (t km^–2^ year^–1^)^b^	Percent chemical
**North side of canal**						
Gatun	115	317	13.7	12.2	715	1.7
Boqueron	91.1	313	14.6	5.46	1,483	0.4
Pequini	145	300	13.5	19.3	1,494	1.2
Chagres	407	463	16.1	10.8	1,413	0.8
Indio Este	80.3	507	14.9	7.18	ND	ND
**South side of canal**						
Ciri Grande	198	315	12.4	2.85	253	1.1
Trinidad	169	312	13.7	4.45	251	1.8
Cano Quebrado	71.2	116	4.56	3.13	124	2.5

*ND* No data.

^a^Long-term cation weathering fluxes based on data from 1998–2015 (n = 18), with the exception of the Cano Quebrado (2005–2015, n = 11) and Indio Este (2008–2015, n = 9).

^b^Long-term suspended sediment fluxes based on data from 2005–2015 (n = 11). No data is available for the Indio Este.

Previous studies have documented a strong relationship between physical and chemical weathering rates worldwide in both large catchments^33^ and SMR watersheds^1–3,10^. Analyzed collectively, our watersheds confirmed this linkage, with 7 of the 11 study years exhibiting a significant positive relationship between cation and sediment fluxes (*r*_avg_ = 0.62, *p*_*avg*_ ≤ 0.17) (Supplementary Tables 5 and 6). However, we observed notable differences in correlation strength when watersheds were analyzed individually over time. For example, we observed positive, yet slightly weaker correlations between these two parameters in the Chagres (*r* = 0.69, *p* = 0.02) and Boqueron (*r* = 0.69, *p* = 0.02) watersheds despite their location on the windward side of Atlantic range where the precipitation rate is corresponding high (> 3,000 mm/year) (Supplementary Table 4) This counterintuitive result could be explained by the fact that these watersheds are characterized by heavy forest cover (> 90%), which has been previously shown to increase infiltration and soil strength and, therefore, reduce surface runoff in tropical watersheds^34,35^.

### Relationship of weathering rates with land use/landcover (LULC) practices

Comparisons between weathering fluxes and LULC practices in tropical SMR watersheds have been limited in scale to date^10,11,13^, due to low temporal resolution geospatial data. However, annual LULC data [forest and mosaic (i.e., forest plus croplands)] measured for the PCW on an annual scale by the ESA Climate Change Initiative (CCI) allowed for their direct comparison with cation values over the 17-year period (The presence of large-scale cropland in only one of the watersheds prevented a comparison with LULC practice). Forest cover was the dominant LULC (> 50%) in all but one of our watersheds and comprised upwards of 95% of LULC in three watersheds (Supplementary Table 7). Our Pearson statistical analysis revealed a positive relationship (*r* ≥ 0.47, *r*_avg_ = 0.63) between percent forest cover and cation fluxes for the entire study period (Supplementary Table 5). Alternatively, we observed mosaic LULC practices to exhibit a strong statistically-significant negative relationship (*r* ≤ − 0.54, *r*_avg_ = − 0.74) with cation fluxes, with 11 of 18 years exhibiting statistical significance.

**Figure 3:**
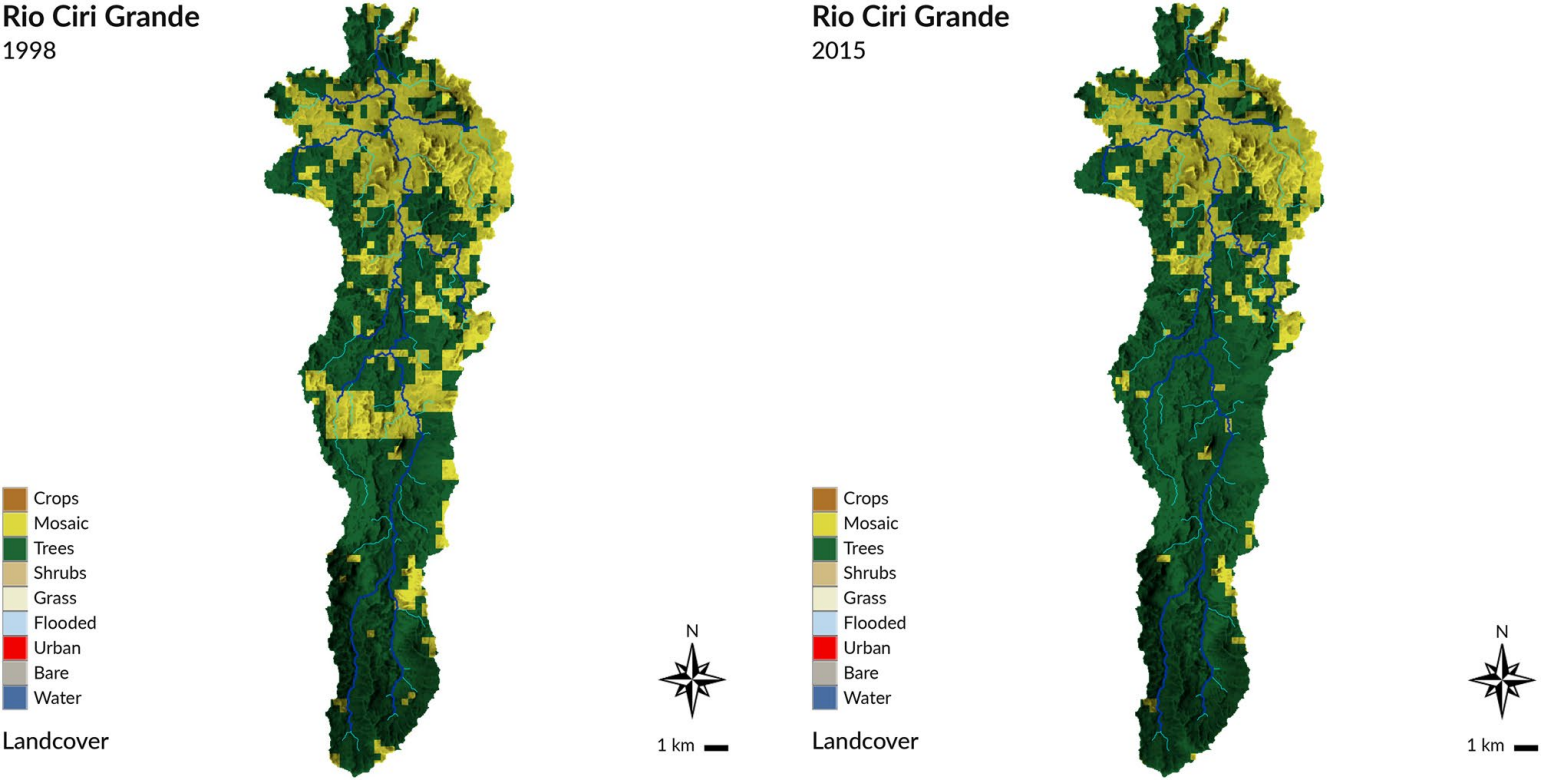
View of landcover change in the Ciri Grande sub-watershed over the course of the study period: a. 1998, and b: 2015. Mosaic landcover decreased by 8.7% over this period and was replaced with forest cover. Landcover data is sourced from the ESA Climate Change Initiative (CCI) Landcover Dataset. Map generated in GRASS GIS 7.4 (GRASS Development Team, 2018; https://grass.osgeo.org/) by the authors.

Our findings are in general agreement with those from a previous isthmus-wide study for Panama based on spot sampling or rivers over a decade^10,11^ and suggests land use practices have fundamentally altered hydrological flow pathways and water residence times in this tropical hydrological system. Previous studies in tropical forested watersheds have observed that some ~ 80–90% of rainfall infiltrates into the soil^34^, where roots can retain water during periods of soil saturation and release it throughout the dry season as baseflow, thus reducing total forest catchment runoff, but increasing flow consistency^34,35^. The concomitant increase in water residence time in contact with fresh mineral surfaces in the soil regolith allows for increased mineral dissolution and solute export. Conversely, soil compaction in agricultural pasture and croplands increase the likelihood of runoff, which is supported by previous studies in the PCW that show mosaic catchments produce 1.8 times more runoff than their forested counterparts^35,36^. Interestingly, the decrease in correlation strength between amount of forest cover and cation fluxes in our study watersheds over time coincided with an increase in percent forest cover in the study catchments over the same period (Fig. 3; Supplementary Tables 5 and 8). This counterintuitive pattern may be attributed to the fact that trees in abandoned mosaic plots, reverting back to forest, have not yet established root networks capable of altering hydrological flow pathways.

### Impact of ENSO on stream discharge and weathering fluxes

We hypothesized that both seasonality and ENSO conditions would influence stream discharge (and by cation and sediment fluxes) and, furthermore, that the two factors would interact. To evaluate the effect of ENSO conditions on stream discharge and weathering fluxes, we used the Oceanic Niño Index (ONI), defined as a 3-month running mean of SST anomalies in the Niño 3.4 region (5º S–58º N, 170º W–120º W)^37^. We justify the use of ONI versus the Southern Oscillation Index (SOI), as previous global compilations studies have observed good agreement between these two indices^28^ and that ONI allows for distinct classification of ENSO events (The National Oceanic and Atmospheric Association classifies El Niño and La Niña events as 3-month ONI running means either < − 0.5 or >  + 0.5 than the long-term average, respectively). We modelled the influence of seasonal periods and ONI on river-discharge and weathering fluxes for the pooled dataset using a mixed-effects model, with seasonal interval (i.e., JFM, etc.) and ONI as fixed effects, and river (i.e., watershed) as a random effect. We accounted for temporal auto-correlation inherent in time-series sampling using an autoregressive AR(1) correlation-structure for residuals. Our model identified a significant interaction of seasonality and ONI on river discharge (p < 0.0001) and cation fluxes (p < 0.0001) and near significance on sediment fluxes (p = 0.056) (Supplementary Table 9). This decoupling between river discharge and sediment flux implies that discharge is not the sole process controlling the sediment flux of PCW rivers. A positive slope-estimate for seasonality is consistent with/reflects the general increase in precipitation from the dry to wet season transition, and corresponding increase in discharge. A negative slope-estimate for ONI suggests less discharge with higher ONI values, which agrees with previous hydrological analyses for the region^23,28,38,39^.

**Figure 4:**
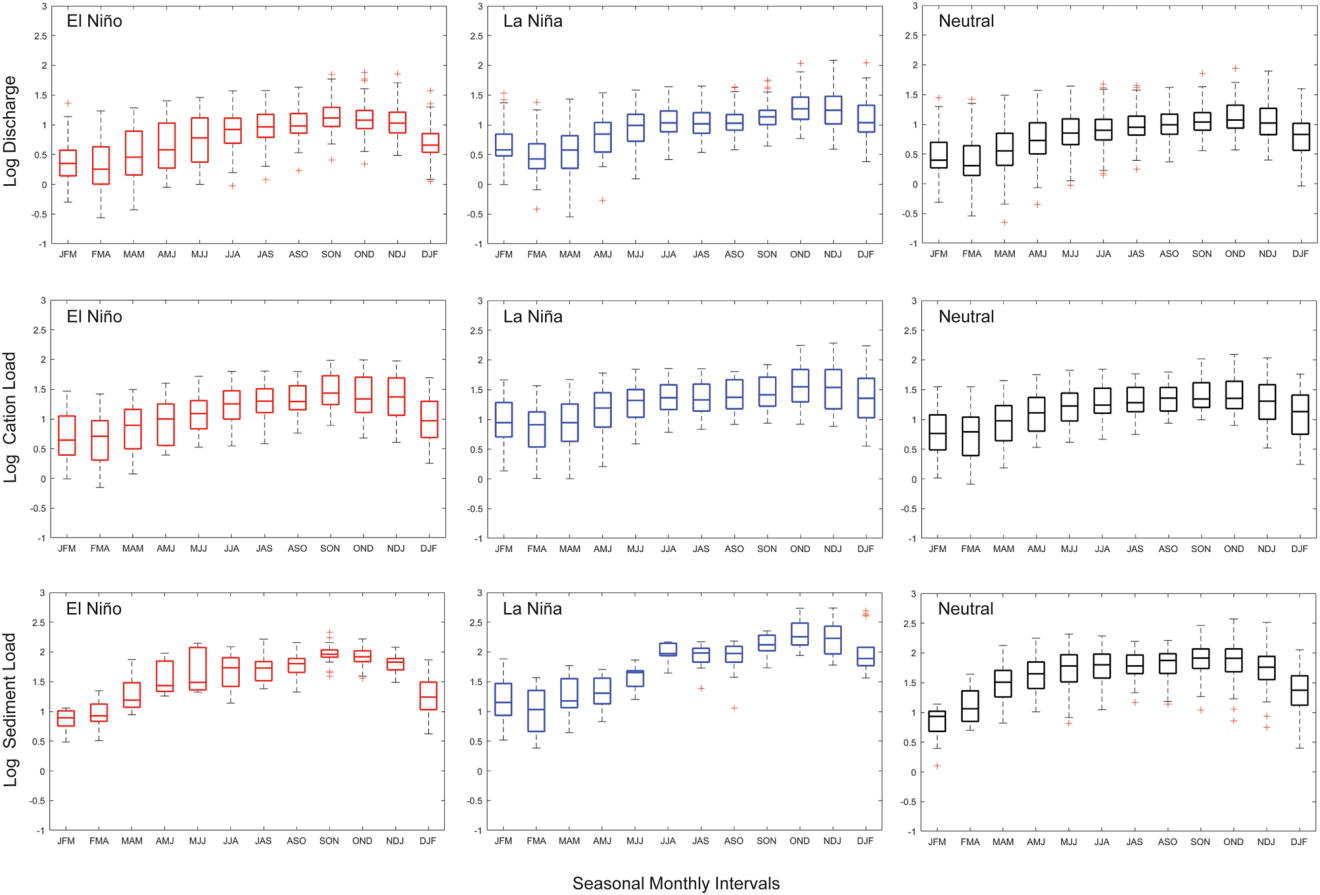
Box and whisker plots of log discharge, cation, and sediment for three-month time intervals for the pooled dataset over the study period (1998–2015). The data is subdivided by ENSO classification: La Niña, neutral, and El Niño. The wet season in Panama extends from May to November.

**Figure 5:**
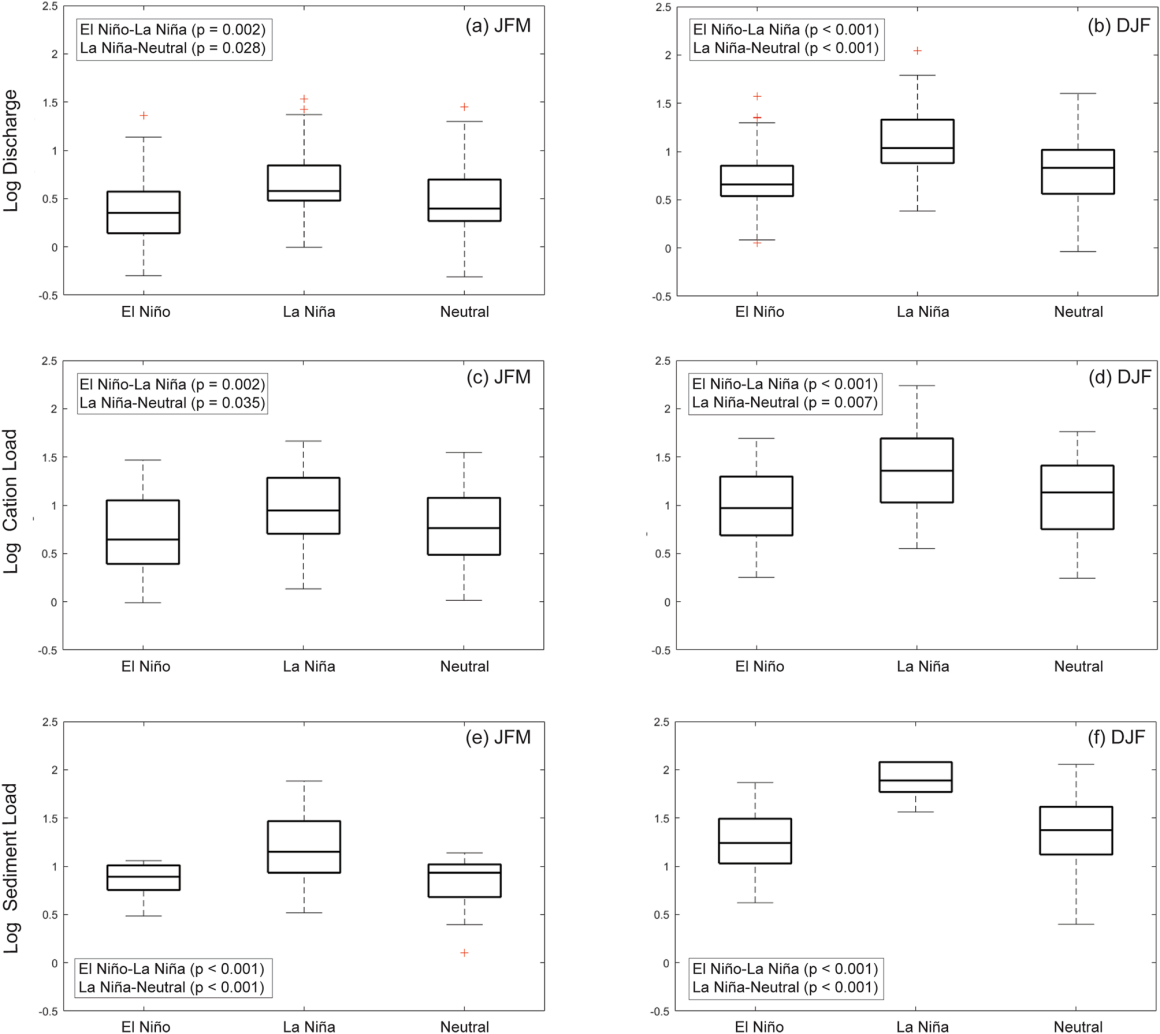
A series of ad-hoc Tukey tests on the pooled discharge, cation, and sediment datasets. For the DJF time interval, significant differences were identified between La Niña and El Niño (p < 0.001) and La Niña and neutral (p ≤ 0.005) discharge and weathering values. For the JFM time interval, significant differences were identified between La Niña and neutral (p ≤ 0.02) and La Nina and El Nino (p = 0.002) discharge and weathering flux values.

Our visual inspection of the PCW dataset identified elevated mean discharge and weathering fluxes for several seasonal timesteps during La Nina conditions (Fig. 4). We then performed a series of one-way ANOVA tests to determine statistically significant differences (p < 0.05) between La Niña, El Niño and neutral discharge as well as weathering fluxes. We confirmed statistical differences only for the December-January–February (DJF) and January–February-March (JFM) periods; with La Niña events exhibiting the highest average values for 9 of the 12 tri-monthly time periods (Fig. 4). Our subsequent series of ad-hoc Tukey tests on the dataset revealed significant differences between La Niña and El Niño (p < 0.001) and La Niña and neutral (p ≤ 0.001) discharge as well as weathering flux values for the DJF period (Fig. 5). Significant differences were also observed between weathering flux values and La Niña and neutral discharge (p ≤ 0.035) and La Nina and El Niño (p ≤ 0.002) discharge values for the JFM period. Previous hydrological studies in Panama and northern South America have identified a positive correlation between monthly average and/or daily maximum precipitation^23,38^, and stream discharge values^23,28,38,39^ with DJF SOI (i.e. drier El Niño and wetter La Niña); the time period when El Niño events typically achieve their maximum. In neighboring Columbia, strong seasonal moisture advection anomalies created by the winds of the “CHOCO jet” have been offered as a possible explanation for a robust regional relationship between precipitation and the DJF SOI time interval^23^, while weaker correlations for March–April-May are potentially explained by the fact that ENSO is either just starting to develop or is declining at that time of the year^23^.

Our observed ENSO-driven deviations in stream discharge have a demonstrable impact on annual cation weathering fluxes. For example, the four highest basin-wide annual average cation yields (Supplementary Table 1; watersheds with 18 year record, only) were recorded in years dominated by strong La Niña events (1999, 2007, 2010, and 2011). This trend was also supported by the sediment yield records for overlapping years (2007, 2010, 2012; Table S2). We also observed that the timing and relative strength of the La Niña event is also an important factor as these same four years exhibited ONI values < 1 through most of the wet season. The only exception to this pattern was in 1998, which was also marked by a strong El Niño event (ONI values > 1) during the first half of the year. Furthermore, a particularly strong La Niña event that occurred during the entirety of the 2010 wet season resulted in average annual cation and sediment fluxes (15–76% and 18%–404%, respectively) that were substantially greater than their respective long-term averages. Exceptionally high rainfall during a 7–8 December 2010 storm, having a return period estimate of 2,000 year^40^, resulted in numerous landslides throughout the PCW^40,41^ and river sediment loads that overwhelmed the Panama City water treatment facilities^40^. High rainfall events associated with La Niña periods have also been linked to an increase in landslides in Columbia and Venezuela^42,43^. Landslides have been previously shown to play a critical role in maintaining the disproportionately high sediment and cation yields in SMR catchments^44,45^, and thus are likely playing a similar role in the PCW.

Conversely, the strong El Niño event observed throughout the 2015 calendar year coincided with our lowest basin-wide average annual cation weathering rate over the 18-year study period. This event was also marked by anomalously high ONI values (i.e. > 2) during the latter half of the year and anecdotal stories regarding water shortages across the canal zone. However, we observed a more variable hydrological response to both neutral and El Niño conditions/events throughout the remainder of the study period, which is supported by our statistical analysis. Previous studies have documented an 8% decrease in rainfall in almost all regions of Panama during 13 El Niño episodes between 1920 and 1983^19^ and below median Lake Gatun inflow in 17 of 20 instances when SST anomalies in the NINO3 region exceed 0.6 ºC^27^. While we did not observe a similar overall hydrological response to El Niño events, this might be in part due to the relative lack of strong El Niño events over our 18-year study period^37^.

ENSO driven deviations in weathering rates are also apparent through comparisons to previous studies utilizing shorter-term data sets. For example, a recent determination of cation weathering rates for the Chagres and Pequini watersheds^10,11^ calculated long-term values 80% and 48% greater, than those of this study. Unsurprisingly, 63% of the spot samples and instantaneous discharge values we used to construct the associated weathering equations for the study were collected during La Niña events. With atmospheric modeling predicting an increase in the frequency of both extreme El Niño^46^ and La Niña events^47^ due to greenhouse gas warming, caution will need to be employed when evaluating future empirically-based weathering studies.

The new findings presented here not only confirm the need for long-term weathering studies in tropical regions, but also suggest that caution should be employed when incorporating data from regions influenced by multi-year or decadal climate patterns into global compilation studies. While much progress has been made over recent decades on the determination of weathering fluxes from high-yielding terrains in the Caribbean^7,9,15^, Central America^10,11^, southeast Asia^8^, and Oceania^2^, these are all regions heavily influenced by ENSO events. Interestingly, these regions have also been shown to play a disproportionate role in the delivery of dissolved^48,49^ and particulate organic carbon^48^ to the global ocean, and by extension, other nutrients such as PO_4_. While ENSO events have been shown to affect vertical mixing and associated upwelling of nutrients in affected ocean areas^50^, our data suggests nearshore locales will also be impacted by concomitant changes in nutrient delivery from the coast. For example, decreased upwelling of nutrients in the eastern Pacific during El Niño periods would be compounded with a corresponding decrease in nutrient export from land, thus exacerbating nutrient limitation in these locales.

## Methods

### Watershed flux calculations

We obtained annual hydrochemical data (1998–2015) for the eight sub-basins of GPCW included in this study from Panama Canal Authority (*Atoridad del Canal de Panamá*) annual hydrologic reports. A specific breakdown of data availability by sub-basin is provided in Supplementary Table 10.

Prior to its use in denudation calculations, streamwater cation concentrations were corrected for sea-salt contribution as follows: non–sea-salt concentration = measured concentration – (sea-salt correction ratio to Cl^–^)*(Cl^–^). Following Murphy and Stallard (2012), we used the following species-to-chloride ratios for this adjustment: Na^+^/Cl^–^ = 0.85251, K^+^/Cl^–^ = 0.01790, Mg^2+^/Cl^–^ = 0.09689, and Ca^2+^/Cl^–^ = 0.01879. We further adjusted for non-silicate contributions of Ca and Mg using volcanic end-member ratios of 0.5 for Ca/Na and 0.5 for Mg/Na, previously established^33^. Our use of these values instead of a continental granitic counterpart is justified by the high Ca/Na and Mg/Na concentrations in both waters and the mafic to andesitic character of igneous rocks across central Panama^10,11^.

Using previously established methodology^10^, we employed a multistep process whereby individual cation concentrations in the data set were first multiplied by the corresponding average daily discharge value in order to produce an instantaneous chemical denudation value. We then prepared x–y plots of instantaneous denudation values with respective discharge to produce specific elemental yield determination equations (Supplementary Table 10). Our approach is supported by high average correlation (*r*^2^) values observed for the instantaneous denudation value-discharge comparisons (Ca = 0.80; Mg = 0.85; Na = 0.82; and K = 0.89). Finally, we substituted daily discharge values over each of the 18 years of record into the equations, and our calculated denudation values were subsequently divided by watershed area to produce annual and long-term estimates of cation weathering rates. For the suspended sediment calculations, we multiplied average daily discharge values by the corresponding average daily suspended sediment concentrations to produce a daily sediment load. Our daily suspended sediment loads were then compiled and subsequently divided by watershed area to produce annual and long-term estimates of suspended sediment yields.

### Hydrological modeling and morphometric analysis of the Greater Panama Canal Watershed in GRASS GIS

The Geographic Resources Analysis Support System (GRASS) GIS dataset used in this study contains topographic, hydrologic, landcover, temperature, and precipitation data and is available on the Open Science Framework at https://osf.io/d5h7s under the CC0 1.0 Universal license. Our topographic data was derived from Japan Aerospace Exploration Agency (JAXA)'s 30 m resolution Advanced Land Observation Satellite (ALOS) Global Digital Surface Model^51,52^. We derived temperature data from the Global Historical Climatology Network (GHCN) and Climate Anomaly Monitoring System (CAMS) global monthly land surface temperature data from January 1948 to April 2018 gridded at 0.5 × 0.5 degree resolution^53^. We derived precipitation data from the Climate Prediction Centers (CPC) Merged Analysis of Precipitation (CMAP) global monthly precipitation data from January 1979 to April 2018 gridded at 2.5 × 2.5 degree resolution^54^. Our landcover data was derived from the ESA Climate Change Initiative (CCI) Landcover Dataset^55^.

GRASS GIS—a free and open source GIS—was used for hydrological modeling and morphometric analysis. For the sake of reproducibility in open science, our geospatial computations in GRASS GIS were automated with Python. The open source code is available under the GNU General Public License (GPL) 2.0 on the Open Science Framework at https://osf.io/bx5y6/ and on GiHub at https://github.com/baharmon/panama_hydrological_modeling.

The ALOS Global Digital Surface Model for the Greater Panama Canal Watershed study area was hydrologically conditioned to reduce noise^56^. Multiple flow direction (MFD) flow accumulation was computed over the hydrologically conditioned digital surface model (DSM) using an A^T^ least-cost search algorithm to traverse depressions and obtacles^57^. We then extracted the stream network from the DSM and flow accumulation, and the stream gage stations were snapped onto the stream network. We derived watershed basin outlets at the stream gages from the flow direction of the stream network. Landcover data from the ESA CCI Landcover Dataset for 1998 through 2015 were reclassified and re-categorized as shown in Supplementary Tables 11 and 12.

We computed topographic, hydrological, and landcover analyses for each basin. The topographic parameters analyzed included elevation, slope, and aspect. Our hydrological parameters included flow accumulation, stream geometry, stream distance, stream order, and stream statistics. Our landcover parameter was the percentage of landcover in each class over the study period. Our topographic, hydrological, and landcover maps were visualized with shaded relief based on the composite of direct illumination derived from topographic relief and diffuse illumination derived from the sky-view factor^58^.

### Statistical analysis

We performed all statistical tests using JMP (Pro version 14.0.0; SAS Institute, Cary, NC). Data which did not meet conditions for normality using a Shapiro–Wilk check test were log transformed prior to analysis. Sample size (*n*) associated with our statistical analyses are provided in the respective supplementary data tables, unless otherwise noted.

We modelled the influence of seasonal period (average tri-monthly interval) and ONI^37^ on the river-discharge (pooled tri-monthly measures n = 1,284), cation flux (n = 1,284), and sediment flux (n = 780) datasets using a linear mixed-effects model (Eqs. 1, 2 and 3):

1 $$ {\text{Q}}_{{{\text{tr}}}} = \, \alpha + \beta_{{1}} {\text{ONI }} + \, \beta_{{2}} {\text{TMI }} + \, \beta_{{3}} {\text{ONI}}*{\text{TMI }} + \, \left( {\mu_{{\text{r}}} + {\text{ error}}} \right), $$

2 $$ Cat_{tr} = \, \alpha + \beta_{{1}} {\text{ONI }} + \, \beta_{{2}} {\text{TMI }} + \, \beta_{{3}} {\text{ONI}}*{\text{TMI }} + \, \left( {\mu_{{\text{r}}} + {\text{ error}}} \right), $$

3 $$ Sed_{tr} = \, \alpha + \beta_{{1}} {\text{ONI }} + \, \beta_{{2}} {\text{TMI }} + \, \beta_{{3}} {\text{ONI}}*{\text{TMI }} + \, \left( {\mu_{{\text{r}}} + {\text{ error}}} \right), $$

where Q_tr,_, *Cat*_*tr*_ , and *Sed*_*tr*_, represents the estimated discharge, cation flux, and sediment flux, respectively, for a particular tri-monthly period over the 18-year dataset; *t*, for a particular river; *r* (Only streams with a full 18 years of data were included in our statistical model for discharge and cation fluxes, which resulted in a total of six river basis. The included sediment record spans eleven years for the same basins). These discharge and weathering flux values were modelled as a function of TMI, a nominal variable indicating the tri-monthly seasonal interval within a year, ONI, a continuous variable, and ONI*TMI representing their interaction. Both visual inspection (graph boxplots of ONI as y-axis and DJF, etc. as x-axis, for the 18-year dataset) and Kendall’s tau suggested little association between ONI and trimonthly interval (*T*_b_ = − 0.0385, *p* = 0.43), therefore we used both as explanatory variables in our statistical analyses. In addition to these fixed effects, µr represents the random effect due to discharge values being associated with the *r*th river and ε is the random error. River was designated as a random factor, as they are a small sample of the total number found in Panama and were not chosen with some explicit comparison in mind.

We accounted for temporal auto-correlation inherent in time-series sampling using an autoregressive AR(1) correlation-structure for residuals. This auto-correlation structure dictates that the smaller the interval between two measures in a time-series, the greater the dependence between them. Multiple model runs were computed evaluating the impact of both different auto-correlation structures, and different random effects, including ARMA(0,0) error structure which does not incorporate any auto-correlation. We used the Akaike information criterion (AIC) to determine the best model, and we report slope estimates for the fixed-effect parameters from this model, calculated using the restricted maximum likelihood method (REML) (Supplementary Table 9).

## Supplementary information

Supplementary file1 (PDF 451 kb)
