# Supplementary material for: Physical controls and ENSO event influence on weathering in the Panama Canal Watershed
Source: Sci Rep. 2020 Jul 2;10:10861. doi: 10.1038/s41598-020-67797-7 (PMC7331659; doi:10.1038/s41598-020-67797-7)
Supplement: Supplementary file 1 — Supplementary file1 (PDF 451 kb) [file 41598_2020_67797_MOESM1_ESM.pdf]

Physical controls and ENSO event influence on weathering in the Panama Canal Watershed

Devin F. Smith, Steven T. Goldsmith, Brendan A. Harmon,

Jorge A. Espinosa, and Russell S. Harmon

Supplementary Information

Table S1: Annual and long-term cation (Ca+Mg+Na+K) yields (in t/km^2/yr) for the PCW watersheds (1998-2015)

Watershed	Area upstream of sampling location (km^2)	1998	1999	2000	2001	2002	2003	2004	2005	2006	2007	2008	2009	2010	2011	2012	2013	2014	2015	Long-term average	std. dev.	CV
North side of canal																						
Gatun	115	11.6	18.8	13.3	9.14	9.41	11.7	12.9	8.41	11.8	14.3	10.4	12.6	21.4	16.6	12.7	8.74	9.22	5.81	12.2	3.74	30.8
Boqueron	91.1	4.29	7.18	5.65	4.49	5.03	4.41	6.75	4.34	5.34	6.06	4.43	6.80	8.03	6.03	6.20	4.88	4.24	4.11	5.46	1.15	21.1
Pequini	145	16.9	24.8	19.6	16.3	19.6	16.3	20.6	14.8	21.7	22.9	16.3	21.7	26.0	22.0	21.3	15.7	15.2	15.4	19.3	3.42	17.7
Chagres	407	8.42	13.6	12.0	7.82	9.34	8.47	11.9	8.39	12.4	12.7	9.93	12.2	13.9	13.6	12.9	9.72	8.16	8.20	10.8	2.16	20.1
Indio Este	80.3											7.57	7.25	9.75	10.6	8.42	6.31	4.03	3.55	7.18	2.33	32.5
South side of canal																						
Ciri Grande	198	1.88	3.85	2.70	2.30	3.31	3.49	2.37	2.20	3.35	3.59	2.55	2.30	3.65	3.57	3.91	2.31	2.18	1.74	2.85	0.71	24.9
Trinidad	169	3.00	5.62	4.25	2.96	4.77	5.81	4.05	3.66	5.30	5.48	3.93	4.10	5.75	5.71	6.57	3.69	3.08	2.37	4.45	1.18	26.6
Cano Quebrado	71.2								2.54	3.10	4.88	2.53	2.63	3.61	4.01	3.64	3.61	2.34	1.58	3.13	0.88	28.2
Basin Wide Average		7.68	12.3	9.59	7.16	8.58	8.36	9.77	6.34	9.00	9.98	7.20	8.69	11.5	10.3	9.45	6.87	6.05	5.35	8.16	1.95	25.2

Table S2: Annual and long-term suspended sediment yields (in t/km^2/yr) for the PCW watersheds (2005-1015)

Watershed	Area upstream of sampling location (km^2)	2005	2006	2007	2008	2009	2010	2011	2012	2013	2014	2015	Long-term average	std. dev.	CV
North side of canal															
Gatun	115	99.0	399	645	300	422	3626	1051	617	229	294	181	715	955	134
Boqueron	91.1	209	584	865	299	1049	6680	1638	1958	567	865	1600	1483	1729	117
Pequini	145	53.9	110	1226	502	1008	6705	1818	1804	896	692	1613	1494	1749	117
Chagres	407	77.9	672	1663	233	575	6346	1920	1811	666	455	1120	1413	1673	118
Indio Este	80.3														
South side of canal															
Ciri Grande	198	61.3	400	286	120	102	448	323	672	145	130	91.5	253	184	72.7
Trinidad	169	94.9	331	277	174	185	433	321	539	191	127	83.0	251	138	55.1
Cano Quebrado	71.2	84.6	130	239	67.4	110	145	151	161	161	69.4	38.8	124	54	43.9
Basin Wide Average		97.2	375	743	242	493	3483	1032	1080	408	376	675	819	926	93.9

Table S3: Pearson correlation coefficients for cation flux analysis (individual)

North side of canal

Parameter	Gatun			Boqueron			Pequini			Chagres			Indio Este		
	n =	*r*	*p*	n =	*r*	*p*	n =	*r*	*p*	n =	*r*	*p*	n =	*r*	*p*
Mean annual rainfall	18	**0.9**	**<0.001**	18	**0.74**	**<0.001**	18	**0.78**	**<0.001**	18	**0.76**	**<0.001**	8	**0.91**	**0.002**
Mean annual temperature	18	-0.42	0.08	18	-0.46	0.06	18	-0.43	0.08	18	*-0.48*	*0.05*	8	-0.33	0.22
SS flux	11	**0.91**	**<0.001**	11	*0.69*	*0.02*	11	0.48	0.040	11	*0.69*	*0.02*	n.d.	n.d.	n.d.

South side of canal

Parameter	Ciri Grande			Trinidad			Cano Quebrado		
	n =	*r*	*p*	n =	*r*	*p*	n =	*r*	*p*
Mean annual rainfall	18	**0.68**	**0.002**	18	**0.77**	**<0.001**	11	**0.83**	**0.002**
Mean annual temperature	18	-0.37	0.14	18	-0.35	0.15	11	-0.28	0.31
SS flux	11	**0.91**	**<0.001**	11	**0.9**	**<0.001**	11	**0.97**	**<0.001**

Note: *r* is Pearson correlation coefficient. Values in bold indicate statistical significance level of 99%. Values in itatlics indicate statsitical signicance of 95%

n.d. = no data

Table S4: Pearson correlation coefficients for suspended sediment flux analysis (individual)

	North side of canal											
Parameter	Gatun			Boqueron			Pequini			Chagres		
	n =	*r*	*p*	n =	*r*	*p*	n =	*r*	*p*	n =	*r*	*p*
Mean annual rainfall	11	*0.66*	*0.03*	11	0.2	0.46	11	0.09	0.77	11	0.36	0.28
Mean annual temperature	11	-0.42	0.08	11	-0.5	0.06	11	-0.29	0.24	11	*-0.48*	*0.05*
Cation flux	11	**0.91**	**<0.001**	11	*0.69*	*0.02*	11	0.48	0.040	11	*0.69*	*0.02*

	South side of canal								
Parameter	Ciri Grande			Trinidad			Cano Quebrado		
	n =	*r*	*p*	n =	*r*	*p*	n =	*r*	*p*
Mean annual rainfall	11	0.59	0.06	11	*0.73*	*0.01*	11	*0.83*	*0.002*
Mean annual temperature	11	-0.37	0.14	11	-0.35	0.15	11	-0.28	0.31
Cation flux	11	**0.91**	**<0.001**	11	**0.9**	**<0.001**	11	**0.97**	**<0.001**

Note: *r* is Pearson correlation coefficient. Values in bold indicate statistical significance level of 99%. Values in itatlics indicate statsitical signicance of 95%

Table S5: Pearson correlation coefficients for cation flux analysis (collective)

Time Period	n	Tree cover		Mosaic cover		Sediment flux		Mean elevation		Mean slope		Minimum slope	
		r	*p*	*r*	*p*	*r*	*p*	*r*	*p*	*r*	*p*	*r*	*p*
1998	6	0.74	0.10	-0.72	0.11			0.14	0.78	0.38	0.46	0.38	0.46
1999	6	0.69	0.13	-0.68	0.14			0.14	0.79	0.35	0.49	0.35	0.49
2000	6	0.73	0.10	-0.69	0.13			0.22	0.68	0.44	0.39	0.44	0.39
2001	6	0.74	0.09	-0.75	0.09			0.13	0.81	0.35	0.50	0.35	0.50
2002	6	0.66	0.15	-0.74	0.09			0.11	0.83	0.30	0.56	0.30	0.56
2003	6	0.47	0.35	-0.54	0.26			0.06	0.91	0.21	0.68	0.21	0.68
2004	6	0.76	0.08	-0.74	0.09			0.20	0.71	0.47	0.34	0.47	0.34
2005	7	0.56	0.19	*-0.79*	*0.03*	-0.19	0.68	0.47	0.34	0.50	0.31	0.50	0.31
2006	7	0.61	0.15	*-0.84*	*0.02*	-0.09	0.85	0.36	0.43	0.36	0.40	0.55	0.20
2007	7	0.47	0.29	*-0.78*	*0.04*	*0.79*	*0.04*	0.37	0.47	0.39	0.44	0.39	0.44
2008	8	0.64	0.09	*-0.81*	*0.01*	*0.81*	*0.03*	0.52	0.29	0.55	0.26	0.55	0.26
2009	8	0.65	0.08	*-0.80*	*0.02*	*0.86*	*0.01*	0.47	0.35	0.54	0.26	0.54	0.26
2010	8	0.65	0.08	*-0.72*	*0.04*	*0.84*	*0.02*	0.46	0.35	0.54	0.26	0.54	0.26
2011	8	0.60	0.12	*-0.72*	*0.04*	*0.78*	*0.04*	0.47	0.34	0.50	0.31	0.50	0.31
2012	8	0.65	0.08	*-0.74*	*0.04*	0.64	0.12	0.50	0.31	0.57	0.24	0.57	0.24
2013	8	0.49	0.22	*-0.77*	*0.03*	*0.81*	*0.03*	0.35	0.50	0.38	0.45	0.38	0.45
2014	8	0.55	0.16	*-0.72*	*0.05*	0.74	0.06	0.37	0.47	0.47	0.35	0.47	0.35
2015	8	0.61	0.10	*-0.83*	*0.01*	*0.83*	*0.02*	0.45	0.37	0.54	0.27	0.54	0.27
LTA	8	0.63	0.14	-0.74	0.07	0.62	0.17	0.32	0.54	0.44	0.39	0.45	0.38

Note: *r* is Pearson correlation coefficient. Values in bold indicate statistical significance level of 99%. Values in itatlics indicate statsitical signicance of 95%

Table S6: Pearson correlation coefficients for suspended sediment flux analysis (collective)

Time Period	n	Tree cover		Mosaic cover		Cation flux		Mean elevation		Mean slope	
		r	*p*	*r*	*p*	*r*	*p*	*r*	*p*	*r*	*p*
2005	7	0.07	0.88	0.11	0.64	-0.19	0.68	0.00	1.00	0.20	0.66
2006	7	0.53	0.22	-0.11	0.81	0.38	0.47	0.76	0.05	0.67	0.10
2007	7	0.66	0.11	**-0.97**	**<0.001**	*0.79*	*0.04*	0.69	0.08	0.62	0.14
2008	7	*0.85*	*0.02*	*-0.77*	*0.03*	*0.81*	*0.03*	0.34	0.45	0.55	0.20
2009	7	0.70	0.08	**-0.92**	**0.004**	*0.86*	*0.01*	0.33	0.47	0.52	0.23
2010	7	*0.84*	*0.02*	**-0.93**	**0.003**	*0.84*	*0.02*	0.55	0.20	0.64	0.12
2011	7	*0.84*	*0.02*	**-0.95**	**0.001**	*0.78*	*0.04*	0.63	0.13	0.70	0.09
2012	7	**0.90**	**0.01**	**-0.91**	**0.005**	0.64	0.12	0.62	0.14	0.64	0.90
2013	7	0.59	0.16	**-0.97**	**<0.001**	*0.81*	*0.03*	0.44	0.32	0.50	0.25
2014	7	*0.76*	*0.05*	**-0.94**	**0.002**	0.74	0.06	0.36	0.42	0.55	0.20
2015	7	0.71	0.07	**-0.99**	**<0.001**	*0.83*	*0.02*	0.40	0.38	0.51	0.24
LTA	7	*0.81*	*0.03*	**-0.95**	**0.001**	*0.80*	*0.03*	0.57	0.18	0.65	0.12

Note: *r* is Pearson correlation coefficient. Values in bold indicate statistical significance level of 99%. Values in italics indicate statistical significance of 95%

Table S7: Total area (in km^2 and %) of land use/landcover (LU/LC) categories above each sampling location (1998-2015)

River	Area upstream of sampling location (km^2)	Total area (in km^2) 1998								Total area (in %) 1998							
		**Crops**	**Mosiac**	**Trees**	**Shrubs**	**Grasses**	**Flooded**	**Bare**	**Total**	**Crops**	**Mosiac**	**Trees**	**Shrubs**	**Grasses**	**Flooded**	**Bare**	**Total**
Gatun	115	0.00	24.5	90.2	0.00	0.00	0.00	0.00	115	0.00	21.4	78.6	0.00	0.00	0.00	0.00	100
Boqueron	92.1	0.00	6.89	85.2	0.00	0.00	0.05	0.00	92.1	0.00	7.47	92.5	0.00	0.00	0.06	0.00	100
Pequini	144	0.00	0.00	144	0.00	0.00	0.00	0.00	144	0.00	0.00	100	0.00	0.00	0.00	0.00	100
Chagres	407	0.00	0.81	406	0.00	0.00	0.36	0.17	407	0.00	0.20	99.7	0.00	0.00	0.09	0.04	100
Indio Este	80.2	0.00	0.69	79	0.13	0.00	0.00	0.01	80.2	0.00	0.86	99.0	0.16	0.00	0.00	0.02	100
Ciri Grande	194	0.00	72.5	122	0.00	0.01	0.00	0.00	194	0.00	37.3	62.7	0.00	0.01	0.00	0.00	100
Trinidad	170	0.00	76.8	92.1	0.00	0.57	0.00	0.00	170	0.00	45.3	54.3	0.00	0.34	0.00	0.00	100
Cano Quebrado	65.7	13.6	50.5	1.38	0.00	0.30	0.00	0.00	65.7	20.6	76.8	2.09	0.00	0.45	0.00	0.00	100

River	Area upstream of sampling location (km^2)	Total area (in km^2) 1999								Total area (in %) 1999							
		**Crops**	**Mosiac**	**Trees**	**Shrubs**	**Grasses**	**Flooded**	**Bare**	**Total**	**Crops**	**Mosiac**	**Trees**	**Shrubs**	**Grasses**	**Flooded**	**Bare**	**Total**
Gatun	115	0.00	27	87.3	0.00	0.00	0.00	0.00	115	0.00	23.9	76.1	0.00	0.00	0.00	0.00	100
Boqueron	92.1	0.00	6.89	85.2	0.00	0.00	0.05	0.00	92.1	0.00	7.47	92.5	0.00	0.00	0.06	0.00	100
Pequini	144	0.00	0.00	144	0.00	0.00	0.00	0.00	144	0.00	0.00	100	0.00	0.00	0.00	0.00	100
Chagres	407	0.00	0.81	406	0.00	0.00	0.36	0.17	407	0.00	0.20	99.7	0.00	0.00	0.09	0.04	100
Indio Este	80.2	0.00	0.69	79.4	0.13	0.00	0.00	0.01	80.3	0.00	0.86	98.96	0.16	0.00	0.00	0.02	100
Ciri Grande	194	0.00	73	122	0.00	0.01	0.00	0.00	194	0.00	37.3	62.7	0.00	0.01	0.00	0.00	100
Trinidad	170	0.00	77	92.1	0.00	0.57	0.00	0.00	170	0.00	45.3	54.3	0.00	0.34	0.00	0.00	100
Cano Quebrado	65.7	13.6	50	1.38	0.00	0.30	0.00	0.00	65.7	20.6	76.8	2.09	0.00	0.45	0.00	0.00	100

River	Area upstream of sampling location (km^2)	Total area (in km^2) 2000								Total area (in %) 2000							
		**Crops**	**Mosiac**	**Trees**	**Shrubs**	**Grasses**	**Flooded**	**Bare**	**Total**	**Crops**	**Mosiac**	**Trees**	**Shrubs**	**Grasses**	**Flooded**	**Bare**	**Total**
Gatun	115	0.00	28.2	86.5	0.00	0.00	0.00	0.00	115	0.00	24.6	75.4	0.00	0.00	0.00	0.00	100
Boqueron	92.1	0.00	6.89	85.2	0.00	0.00	0.05	0.00	92.1	0.00	7.47	92.5	0.00	0.00	0.06	0.00	100
Pequini	144	0.00	0.00	144	0.00	0.00	0.00	0.00	144	0.00	0.00	100	0.00	0.00	0.00	0.00	100
Chagres	407	0.00	0.81	406	0.00	0.00	0.36	0.17	407	0.00	0.20	99.7	0.00	0.00	0.09	0.04	100
Indio Este	80.2	0.00	0.69	79.4	0.13	0.00	0.00	0.01	80.2	0.00	0.86	99.0	0.16	0.00	0.00	0.02	100
Ciri Grande	194	0.00	72.5	122	0.00	0.01	0.00	0.00	194	0.00	37.3	62.7	0.00	0.01	0.00	0.00	100
Trinidad	170	0.00	76.8	92.1	0.00	0.57	0.00	0.00	170	0.00	45.3	54.3	0.00	0.34	0.00	0.00	100
Cano Quebrado	65.7	13.6	50.5	1.38	0.00	0.30	0.00	0.00	65.7	20.6	76.8	2.09	0.00	0.45	0.00	0.00	100

Table S7 (cont'd): Total area (in km^2 and %) of land use/landcover (LU/LC) categories above each sampling location (1998-2015)

River	Area upstream of sampling location (km^2)	Total area (in km^2) 2001								Total area (in %) 2001							
		**Crops**	**Mosiac**	**Trees**	**Shrubs**	**Grasses**	**Flooded**	**Bare**	**Total**	**Crops**	**Mosiac**	**Trees**	**Shrubs**	**Grasses**	**Flooded**	**Bare**	**Total**
Gatun	115	0.00	29.0	85.7	0.00	0.00	0.00	0.00	115	0.00	25.3	74.7	0.00	0.00	0.00	0.00	100
Boqueron	92.1	0.00	5.81	86.3	0.00	0.00	0.05	0.00	92.1	0.00	6.30	93.6	0.00	0.00	0.06	0.00	100
Pequini	144	0.00	0.00	144	0.00	0.00	0.00	0.00	144	0.00	0.00	100	0.00	0.00	0.00	0.00	100
Chagres	407	0.00	0.81	406	0.00	0.00	0.36	0.17	407	0.00	0.20	99.7	0.00	0.00	0.09	0.04	100
Indio Este	80.2	0.00	0.69	79.4	0.13	0.00	0.00	0.01	80.2	0.00	0.86	99.0	0.16	0.00	0.00	0.02	100
Ciri Grande	194	0.00	72.5	122	0.00	0.01	0.00	0.00	194	0.00	37.3	62.7	0.00	0.01	0.00	0.00	100
Trinidad	170	0.00	75.5	93.5	0.00	0.57	0.00	0.00	170	0.00	44.5	55.1	0.00	0.34	0.00	0.00	100
Cano Quebrado	65.7	13.6	50.5	1.38	0.00	0.30	0.00	0.00	65.7	20.6	76.8	2.09	0.00	0.45	0.00	0.00	100

River	Area upstream of sampling location (km^2)	Total area (in km^2) 2002								Total area (in %) 2002							
		**Crops**	**Mosiac**	**Trees**	**Shrubs**	**Grasses**	**Flooded**	**Bare**	**Total**	**Crops**	**Mosiac**	**Trees**	**Shrubs**	**Grasses**	**Flooded**	**Bare**	**Total**
Gatun	115	0.00	29.2	85.5	0.00	0.00	0.00	0.00	115	0.00	25.5	74.5	0.00	0.00	0.00	0.00	100
Boqueron	92.1	0.00	4.46	87.6	0.00	0.00	0.05	0.00	92.1	0.00	4.84	95.1	0.00	0.00	0.06	0.00	100
Pequini	144	0.00	0.00	144	0.00	0.00	0.00	0.00	144	0.00	0.00	100	0.00	0.00	0.00	0.00	100
Chagres	407	0.00	0.81	406	0.00	0.00	0.36	0.17	407	0.00	0.20	99.7	0.00	0.00	0.09	0.04	100
Indio Este	80.2	0.00	0.69	79.4	0.13	0.00	0.00	0.01	80.2	0.00	0.86	99.0	0.16	0.00	0.00	0.02	100
Ciri Grande	194	0.00	72.5	122	0.00	0.01	0.00	0.00	194	0.00	37.3	62.7	0.00	0.01	0.00	0.00	100
Trinidad	170	0.00	75.5	93.5	0.00	0.57	0.00	0.00	170	0.00	44.5	55.1	0.00	0.34	0.00	0.00	100
Cano Quebrado	65.7	13.6	50.5	1.38	0.00	0.30	0.00	0.00	65.7	20.6	76.8	2.09	0.00	0.45	0.00	0.00	100

River	Area upstream of sampling location (km^2)	Total area (in km^2) 2003								Total area (in %) 2003							
		**Crops**	**Mosiac**	**Trees**	**Shrubs**	**Grasses**	**Flooded**	**Bare**	**Total**	**Crops**	**Mosiac**	**Trees**	**Shrubs**	**Grasses**	**Flooded**	**Bare**	**Total**
Gatun	115	0.00	29.2	85.5	0.00	0.00	0.00	0.00	115	0.00	25.5	74.5	0.00	0.00	0.00	0.00	100
Boqueron	92.1	0.00	4.46	87.6	0.00	0.00	0.05	0.00	92.1	0.00	4.84	95.1	0.00	0.00	0.06	0.00	100
Pequini	144	0.00	0.00	144	0.00	0.00	0.00	0.00	144	0.00	0.00	100	0.00	0.00	0.00	0.00	100
Chagres	407	0.00	0.81	406	0.00	0.00	0.36	0.17	407	0.00	0.20	99.7	0.00	0.00	0.09	0.04	100
Indio Este	80.2	0.00	0.69	79.4	0.13	0.00	0.00	0.01	80.2	0.00	0.86	99.0	0.16	0.00	0.00	0.02	100
Ciri Grande	194	0.00	72.5	122	0.00	0.01	0.00	0.00	194	0.00	37.3	62.7	0.00	0.01	0.00	0.00	100
Trinidad	170	0.00	75.5	93.5	0.00	0.57	0.00	0.00	170	0.00	44.5	55.1	0.00	0.34	0.00	0.00	100
Cano Quebrado	65.7	13.6	49.7	2.19	0.00	0.30	0.00	0.00	65.7	20.6	75.6	3.33	0.00	0.45	0.00	0.00	100

Table S7 (cont'd): Total area (in km^2 and %) of land use/landcover (LU/LC) categories above each sampling location (1998-2015)

River	Area upstream of sampling location (km^2)	Total area (in km^2) 2004								Total area (in %) 2004							
		**Crops**	**Mosiac**	**Trees**	**Shrubs**	**Grasses**	**Flooded**	**Bare**	**Total**	**Crops**	**Mosiac**	**Trees**	**Shrubs**	**Grasses**	**Flooded**	**Bare**	**Total**
Gatun	115	0.00	28.7	86.1	0.00	0.00	0.00	0.00	115	0.00	25.0	75.0	0.00	0.00	0.00	0.00	100
Boqueron	92.1	0.00	3.83	88.1	0.00	0.00	0.23	0.00	92.1	0.00	4.15	95.6	0.00	0.00	0.25	0.00	100
Pequini	144	0.00	0.00	144	0.00	0.00	0.00	0.00	144	0.00	0.00	100	0.00	0.00	0.00	0.00	100
Chagres	407	0.00	0.81	406	0.00	0.00	0.36	0.17	407	0.00	0.20	99.7	0.00	0.00	0.09	0.04	100
Indio Este	80.2	0.00	0.69	79	0.13	0.00	0.00	0.01	80.2	0.00	0.86	99.0	0.16	0.00	0.00	0.02	100
Ciri Grande	194	0.00	72.5	122	0.00	0.01	0.00	0.00	194	0.00	37.3	62.7	0.00	0.01	0.00	0.00	100
Trinidad	170	0.00	72.5	93.5	0.00	0.57	0.00	0.00	167	0.00	43.5	56.1	0.00	0.34	0.00	0.00	100
Cano Quebrado	65.7	13.6	49.0	2.81	0.00	0.30	0.05	0.00	65.7	20.6	74.6	4.28	0.00	0.45	0.07	0.00	100

River	Area upstream of sampling location (km^2)	Total area (in km^2) 2005								Total area (in %) 2005							
		**Crops**	**Mosiac**	**Trees**	**Shrubs**	**Grasses**	**Flooded**	**Bare**	**Total**	**Crops**	**Mosiac**	**Trees**	**Shrubs**	**Grasses**	**Flooded**	**Bare**	**Total**
Gatun	115	0.00	28.7	86.1	0.00	0.00	0.00	0.00	115	0.00	25.0	75.0	0.00	0.00	0.00	0.00	100
Boqueron	92.1	0.00	3.83	88.1	0.00	0.00	0.23	0.00	92.1	0.00	4.15	95.6	0.00	0.00	0.25	0.00	100
Pequini	144	0.00	0.00	144	0.00	0.00	0.00	0.00	144	0.00	0.00	100	0.00	0.00	0.00	0.00	100
Chagres	407	0.00	0.81	406	0.00	0.00	0.36	0.17	407	0.00	0.20	99.7	0.00	0.00	0.09	0.04	100
Indio Este	80.2	0.00	0.69	79.4	0.13	0.00	0.00	0.01	80.2	0.00	0.86	99.0	0.16	0.00	0.00	0.02	100
Ciri Grande	194	0.00	72.5	122	0.00	0.01	0.00	0.00	194	0.00	37.3	62.7	0.00	0.01	0.00	0.00	100
Trinidad	170	0.00	75.5	93.5	0.00	0.57	0.00	0.00	170	0.00	44.5	55.1	0.00	0.34	0.00	0.00	100
Cano Quebrado	65.7	13.6	49.0	2.81	0.00	0.30	0.05	0.00	65.7	20.6	74.6	4.28	0.00	0.45	0.07	0.00	100

River	Area upstream of sampling location (km^2)	Total area (in km^2) 2006								Total area (in %) 2006							
		**Crops**	**Mosiac**	**Trees**	**Shrubs**	**Grasses**	**Flooded**	**Bare**	**Total**	**Crops**	**Mosiac**	**Trees**	**Shrubs**	**Grasses**	**Flooded**	**Bare**	**Total**
Gatun	115	0.00	24.7	90.1	0.00	0.00	0.00	0.00	115	0.00	21.5	78.5	0.00	0.00	0.00	0.00	100
Boqueron	92.1	0.00	3.83	88.1	0.00	0.00	0.23	0.00	92.1	0.00	4.15	95.6	0.00	0.00	0.25	0.00	100
Pequini	144	0.00	0.00	144	0.00	0.00	0.00	0.00	144	0.00	0.00	100	0.00	0.00	0.00	0.00	100
Chagres	407	0.00	0.81	406	0.00	0.00	0.36	0.17	407	0.00	0.20	99.7	0.00	0.00	0.09	0.04	100
Indio Este	80.2	0.00	0.69	79.4	0.13	0.00	0.00	0.01	80.2	0.00	0.86	99.0	0.16	0.00	0.00	0.02	100
Ciri Grande	194	0.00	72.5	122	0.00	0.01	0.00	0.00	194	0.00	37.3	62.7	0.00	0.01	0.00	0.00	100
Trinidad	170	0.00	75.5	93.5	0.00	0.57	0.00	0.00	170	0.00	44.5	55.1	0.00	0.34	0.00	0.00	100
Cano Quebrado	65.7	13.6	49.0	2.81	0.00	0.30	0.05	0.00	65.7	20.6	74.6	4.28	0.00	0.45	0.07	0.00	100

Table S7 (cont'd): Total area (in km^2 and %) of land use/landcover (LU/LC) categories above each sampling location (1998-2015)

River	Area upstream of sampling location (km^2)	Total area (in km^2) 2007								Total area (in %) 2007							
		**Crops**	**Mosiac**	**Trees**	**Shrubs**	**Grasses**	**Flooded**	**Bare**	**Total**	**Crops**	**Mosiac**	**Trees**	**Shrubs**	**Grasses**	**Flooded**	**Bare**	**Total**
Gatun	115	0.00	18.0	96.7	0.00	0.00	0.00	0.00	115	0.00	15.7	84.3	0.00	0.00	0.00	0.00	100
Boqueron	92.1	0.00	3.83	88.1	0.00	0.00	0.23	0.00	92.1	0.00	4.15	95.6	0.00	0.00	0.25	0.00	100
Pequini	144	0.00	0.00	144	0.00	0.00	0.00	0.00	144	0.00	0.00	100	0.00	0.00	0.00	0.00	100
Chagres	407	0.00	0.81	406	0.00	0.00	0.36	0.17	407	0.00	0.20	99.7	0.00	0.00	0.09	0.04	100
Indio Este	80.2	0.00	0.69	79.4	0.13	0.00	0.00	0.01	80.2	0.00	0.86	99.0	0.16	0.00	0.00	0.02	100
Ciri Grande	194	0.00	72.5	122	0.00	0.01	0.00	0.00	194	0.00	37.3	62.7	0.00	0.01	0.00	0.00	100
Trinidad	170	0.00	75.5	93.5	0.00	0.57	0.00	0.00	170	0.00	44.5	55.1	0.00	0.34	0.00	0.00	100
Cano Quebrado	65.7	14	49.0	2.81	0.00	0.30	0.05	0.00	65.7	20.6	74.6	4.28	0.00	0.45	0.07	0.00	100

River	Area upstream of sampling location (km^2)	Total area (in km^2) 2008								Total area (in %) 2008							
		**Crops**	**Mosiac**	**Trees**	**Shrubs**	**Grasses**	**Flooded**	**Bare**	**Total**	**Crops**	**Mosiac**	**Trees**	**Shrubs**	**Grasses**	**Flooded**	**Bare**	**Total**
Gatun	115	0.00	17.1	97.6	0.00	0.00	0.00	0.00	115	0.00	14.9	85.1	0.00	0.00	0.00	0.00	100
Boqueron	92.1	0.00	3.83	88.1	0.00	0.00	0.23	0.00	92.1	0.00	4.15	95.6	0.00	0.00	0.25	0.00	100
Pequini	144	0.00	0.00	144	0.00	0.00	0.00	0.00	144	0.00	0.00	100.00	0.00	0.00	0.00	0.00	100
Chagres	407	0.00	0.81	406	0.00	0.00	0.36	0.17	407	0.00	0.20	99.7	0.00	0.00	0.09	0.04	100
Indio Este	80.2	0.00	0.69	79.4	0.13	0.00	0.00	0.01	80.2	0.00	0.86	99.0	0.16	0.00	0.00	0.02	100
Ciri Grande	194	0.00	72.0	122	0.00	0.01	0.00	0.00	194	0.00	37.0	63.0	0.00	0.01	0.00	0.00	100
Trinidad	170	0.00	75.5	93.5	0.00	0.57	0.00	0.00	170	0.00	44.5	55.1	0.00	0.34	0.00	0.00	100
Cano Quebrado	65.7	12.9	47.4	5.02	0.00	0.30	0.05	0.00	65.7	19.6	72.2	7.63	0.00	0.45	0.07	0.00	100

River	Area upstream of sampling location (km^2)	Total area (in km^2) 2009								Total area (in %) 2009							
		**Crops**	**Mosiac**	**Trees**	**Shrubs**	**Grasses**	**Flooded**	**Bare**	**Total**	**Crops**	**Mosiac**	**Trees**	**Shrubs**	**Grasses**	**Flooded**	**Bare**	**Total**
Gatun	115	0.00	15.3	99.4	0.00	0.00	0.00	0.00	115	0.00	13.4	86.6	0.00	0.00	0.00	0.00	100
Boqueron	92.1	0.00	1.20	90.7	0.00	0.00	0.23	0.00	92.1	0.00	1.30	98.4	0.00	0.00	0.25	0.00	100
Pequini	144	0.00	0.00	144	0.00	0.00	0.00	0.00	144	0.00	0.00	100	0.00	0.00	0.00	0.00	100
Chagres	407	0.00	0.81	406	0.00	0.00	0.36	0.17	407	0.00	0.20	99.7	0.00	0.00	0.09	0.04	100
Indio Este	80.2	0.00	0.69	79.4	0.13	0.00	0.00	0.01	80.2	0.00	0.86	99.0	0.16	0.00	0.00	0.02	100
Ciri Grande	194	0.00	71.0	123.4	0.00	0.01	0.00	0.00	194	0.00	36.5	63.5	0.00	0.01	0.00	0.00	100
Trinidad	170	0.00	75.5	93.5	0.00	0.57	0.00	0.00	170	0.00	44.5	55.1	0.00	0.34	0.00	0.00	100
Cano Quebrado	65.7	12.9	46.4	6.07	0.00	0.30	0.05	0.00	65.7	19.6	70.6	9.24	0.00	0.45	0.07	0.00	100

Table S7 (cont'd): Total area (in km^2 and %) of land use/landcover (LU/LC) categories above each sampling location (1998-2015)

River	Area upstream of sampling location (km^2)	Total area (in km^2) 2010								Total area (in %) 2010							
		**Crops**	**Mosiac**	**Trees**	**Shrubs**	**Grasses**	**Flooded**	**Bare**	**Total**	**Crops**	**Mosiac**	**Trees**	**Shrubs**	**Grasses**	**Flooded**	**Bare**	**Total**
Gatun	115	0.00	14.6	100	0.00	0.00	0.00	0.00	115	0.00	12.7	87.3	0.00	0.00	0.00	0.00	100
Boqueron	92.1	0.00	1.20	90.7	0.00	0.00	0.23	0.00	92.1	0.00	1.30	98.4	0.00	0.00	0.25	0.00	100
Pequini	144	0.00	0.00	144	0.00	0.00	0.00	0.00	144	0.00	0.00	100	0.00	0.00	0.00	0.00	100
Chagres	407	0.00	0.81	406	0.00	0.00	0.36	0.17	407	0.00	0.20	99.7	0.00	0.00	0.09	0.04	100
Indio Este	80.2	0.00	0.69	79.4	0.13	0.00	0.00	0.01	80.2	0.00	0.86	99.0	0.16	0.00	0.00	0.02	100
Ciri Grande	194	0.00	67.4	127	0.00	0.01	0.00	0.00	194	0.00	34.7	65.3	0.00	0.01	0.00	0.00	100
Trinidad	170	0.00	71.3	97.7	0.00	0.57	0.00	0.00	170	0.00	42.0	57.6	0.00	0.34	0.00	0.00	100
Cano Quebrado	65.7	12.4	45.1	7.78	0.00	0.30	0.05	0.00	65.7	18.9	68.7	11.8	0.00	0.45	0.07	0.00	100

River	Area upstream of sampling location (km^2)	Total area (in km^2) 2011								Total area (in %) 2011							
		**Crops**	**Mosiac**	**Trees**	**Shrubs**	**Grasses**	**Flooded**	**Bare**	**Total**	**Crops**	**Mosiac**	**Trees**	**Shrubs**	**Grasses**	**Flooded**	**Bare**	**Total**
Gatun	115	0.00	14.2	101	0.00	0.00	0.00	0.00	115	0.00	12.4	87.6	0.00	0.00	0.00	0.00	100
Boqueron	92.1	0.00	1.20	90.8	0.00	0.00	0.23	0.00	92.2	0.00	1.30	98.4	0.00	0.00	0.25	0.00	100
Pequini	144	0.00	0.00	144	0.00	0.00	0.00	0.00	144	0.00	0.00	100	0.00	0.00	0.00	0.00	100
Chagres	407	0.00	0.81	406	0.00	0.00	0.36	0.17	407	0.00	0.20	99.7	0.00	0.00	0.09	0.04	100
Indio Este	80.2	0.00	0.69	79.4	0.13	0.00	0.00	0.01	80.2	0.00	0.86	99.0	0.16	0.00	0.00	0.02	100
Ciri Grande	194	0.00	65.3	129	0.00	0.01	0.00	0.00	194	0.00	33.6	66.4	0.00	0.01	0.00	0.00	100
Trinidad	170	0.00	69.5	99.5	0.00	0.57	0.00	0.00	170	0.00	41.0	58.7	0.00	0.34	0.00	0.00	100
Cano Quebrado	65.7	12.4	45.1	7.78	0.00	0.30	0.05	0.00	65.7	18.9	68.7	11.8	0.00	0.45	0.07	0.00	100

River	Area upstream of sampling location (km^2)	Total area (in km^2) 2012								Total area (in %) 2012							
		**Crops**	**Mosiac**	**Trees**	**Shrubs**	**Grasses**	**Flooded**	**Bare**	**Total**	**Crops**	**Mosiac**	**Trees**	**Shrubs**	**Grasses**	**Flooded**	**Bare**	**Total**
Gatun	115	0.00	14.2	101	0.00	0.00	0.00	0.00	115	0.00	12.4	87.6	0.00	0.00	0.00	0.00	100.00
Boqueron	92.1	0.00	1.20	90.8	0.00	0.00	0.23	0.00	92.2	0.00	1.30	98.4	0.00	0.00	0.25	0.00	100.00
Pequini	144	0.00	0.00	144	0.00	0.00	0.00	0.00	144	0.00	0.00	100	0.00	0.00	0.00	0.00	100.00
Chagres	407	0.00	0.81	406	0.00	0.00	0.36	0.17	407	0.00	0.20	99.7	0.00	0.00	0.09	0.04	100.00
Indio Este	80.2	0.00	0.69	79.4	0.13	0.00	0.00	0.01	80.2	0.00	0.86	99.0	0.16	0.00	0.00	0.02	100.00
Ciri Grande	194	0.00	65.3	129	0.00	0.01	0.00	0.00	194	0.00	33.6	66.4	0.00	0.01	0.00	0.00	100.00
Trinidad	170	0.00	68.7	100	0.00	0.57	0.00	0.00	170	0.00	40.5	59.1	0.00	0.34	0.00	0.00	100.00
Cano Quebrado	65.7	12.4	45.1	7.80	0.00	0.30	0.05	0.00	65.7	18.9	68.7	11.9	0.00	0.45	0.07	0.00	100.00

Table S7 (cont'd): Total area (in km^2 and %) of land use/landcover (LU/LC) categories above each sampling location (1998-2015)

River	Area upstream of sampling location (km^2)	Total area (in km^2) 2013								Total area (in %) 2013							
		**Crops**	**Mosiac**	**Trees**	**Shrubs**	**Grasses**	**Flooded**	**Bare**	**Total**	**Crops**	**Mosiac**	**Trees**	**Shrubs**	**Grasses**	**Flooded**	**Bare**	**Total**
Gatun	115	0.00	12.6	102	0.00	0.00	0.00	0.00	115	0.00	11.0	89.0	0.00	0.00	0.00	0.00	100
Boqueron	92.1	0.00	1.10	90.8	0.00	0.00	0.23	0.00	92.1	0.00	1.20	98.5	0.00	0.00	0.25	0.00	100
Pequini	144	0.00	0.00	144	0.00	0.00	0.00	0.00	144	0.00	0.00	100	0.00	0.00	0.00	0.00	100
Chagres	407	0.00	0.81	406	0.00	0.00	0.36	0.17	407	0.00	0.20	99.7	0.00	0.00	0.09	0.04	100
Indio Este	80.2	0.00	0.69	79.4	0.13	0.00	0.00	0.01	80.2	0.00	0.86	99.0	0.16	0.00	0.00	0.02	100
Ciri Grande	194	0.00	62.0	132	0.00	0.01	0.00	0.00	194	0.00	31.9	68.1	0.00	0.01	0.00	0.00	100
Trinidad	170	0.00	68.7	100	0.00	0.57	0.00	0.00	170	0.00	40.5	59.1	0.00	0.34	0.00	0.00	100
Cano Quebrado	65.7	12.4	45.1	7.80	0.00	0.30	0.05	0.00	65.7	18.9	68.7	11.9	0.00	0.45	0.07	0.00	100

River	Area upstream of sampling location (km^2)	Total area (in km^2) 2014								Total area (in %) 2014							
		**Crops**	**Mosiac**	**Trees**	**Shrubs**	**Grasses**	**Flooded**	**Bare**	**Total**	**Crops**	**Mosiac**	**Trees**	**Shrubs**	**Grasses**	**Flooded**	**Bare**	**Total**
Gatun	115	0.00	12.6	102	0.00	0.00	0.00	0.00	115	0.00	11.0	89.0	0.00	0.00	0.00	0.00	100
Boqueron	92.1	0.00	1.10	90.8	0.00	0.00	0.23	0.00	92.1	0.00	1.20	98.5	0.00	0.00	0.25	0.00	100
Pequini	144	0.00	0.00	144	0.00	0.00	0.00	0.00	144	0.00	0.00	100	0.00	0.00	0.00	0.00	100
Chagres	407	0.00	0.81	406	0.00	0.00	0.36	0.17	407	0.00	0.20	99.7	0.00	0.00	0.09	0.04	100
Indio Este	80.2	0.00	0.69	79.4	0.13	0.00	0.00	0.01	80.2	0.00	0.86	99.0	0.16	0.00	0.00	0.02	100
Ciri Grande	194	0.00	55.6	139	0.00	0.01	0.00	0.00	194	0.00	28.6	71.4	0.00	0.01	0.00	0.00	100
Trinidad	170	0.00	56.8	112	0.00	0.57	0.00	0.00	170	0.00	33.5	66.2	0.00	0.34	0.00	0.00	100
Cano Quebrado	65.7	12.4	45.1	7.80	0.00	0.30	0.05	0.00	65.7	18.9	68.7	11.9	0.00	0.45	0.07	0.00	100

River	Area upstream of sampling location (km^2)	Total area (in km^2) 2015								Total area (in %) 2015							
		**Crops**	**Mosiac**	**Trees**	**Shrubs**	**Grasses**	**Flooded**	**Bare**	**Total**	**Crops**	**Mosiac**	**Trees**	**Shrubs**	**Grasses**	**Flooded**	**Bare**	**Total**
Gatun	115	0.00	12.6	102	0.00	0.00	0.00	0.00	115	0.00	11.0	89.0	0.00	0.00	0.00	0.00	100
Boqueron	92.1	0.00	1.10	90.8	0.00	0.00	0.23	0.00	92.1	0.00	1.20	98.5	0.00	0.00	0.25	0.00	100
Pequini	144	0.00	0.00	144	0.00	0.00	0.00	0.00	144	0.00	0.00	100	0.00	0.00	0.00	0.00	100
Chagres	407	0.00	0.81	406	0.00	0.00	0.36	0.17	407	0.00	0.20	99.7	0.00	0.00	0.09	0.04	100
Indio Este	80.2	0.00	0.69	79.4	0.13	0.00	0.00	0.01	80.2	0.00	0.86	99.0	0.16	0.00	0.00	0.02	100
Ciri Grande	194	0.00	55.6	139	0.00	0.01	0.00	0.00	194	0.00	28.6	71.4	0.00	0.01	0.00	0.00	100
Trinidad	170	0.00	56.8	112	0.00	0.57	0.00	0.00	170	0.00	33.5	66.2	0.00	0.34	0.00	0.00	100
Cano Quebrado	65.7	12.4	45.1	7.80	0.00	0.30	0.05	0.00	65.7	18.9	68.7	11.9	0.00	0.45	0.07	0.00	100

Table S7 (cont'd): Total area (in km^2 and %) of land use/landcover (LU/LC) categories above each sampling location (1998-2015)

River	Area upstream of sampling location (km^2)	Total area (in km^2) LTA								Total area (in %) LTA							
		**Crops**	**Mosiac**	**Trees**	**Shrubs**	**Grasses**	**Flooded**	**Bare**	**Total**	**Crops**	**Mosiac**	**Trees**	**Shrubs**	**Grasses**	**Flooded**	**Bare**	**Total**
Gatun	115	0.00	21.2	93.6	0.00	0.00	0.00	0.00	115	0.00	18.4	81.6	0.00	0.00	0.00	0.00	100
Boqueron	92.1	0.00	3.48	88.5	0.00	0.00	0.17	0.00	92.1	0.00	3.77	96.0	0.00	0.00	0.19	0.00	100
Pequini	144	0.00	0.00	144	0.00	0.00	0.00	0.00	144	0.00	0.00	100	0.00	0.00	0.00	0.00	100
Chagres	407	0.00	0.81	406	0.00	0.00	0.36	0.17	407	0.00	0.20	99.7	0.00	0.00	0.09	0.04	100
Indio Este	80.2	0.00	0.69	79.4	0.13	0.00	0.00	0.01	80.2	0.00	0.86	99.0	0.16	0.00	0.00	0.02	100
Ciri Grande	194	0.00	68.9	126	0.00	0.01	0.00	0.00	194	0.00	35.4	64.6	0.00	0.01	0.00	0.00	100
Trinidad	170	0.00	72.2	96.7	0.00	0.57	0.00	0.00	169	0.00	42.6	57.1	0.00	0.34	0.00	0.00	100
Cano Quebrado	65.7	13.1	47.9	4.34	0.00	0.30	0.03	0.00	65.7	20.0	72.9	6.61	0.00	0.45	0.05	0.00	100

Table S8: Percent change in LULC for PCW watersheds from 1998-2015

River	Crops	Mosiac	Trees	Shrubs	Grasses	Flooded	Bare
			North side of canal				
Gatun		-10.4%	10.4%				
Boqueron		-6.3%	6.1%			0.2%	
Pequini							
Chagres							
Indio Este							
			South side of canal				
Ciri Grande		-8.7%	8.7%				
Trinidad		-11.8%	11.8%				
Cano Quebrado	-1.7%	-8.2%	9.8%				

Table S9: Mixed-model analysis evaluating discharge, cation flux, sediment flux, as a function of both season and ENSO conditions

AiC Value	Parameter	Parameter slope esitmate β	DF	t-ratio	*p* -value
Discharge					
	Intercept	0.571	5.1	4.74	0.0049
-466.1773	TMI[1]	0.0578	1275	35.74	<0.001
	ONI[2]	-0.0672	1275	-8.8	<0.001
	TMI * ONI	0.00069	1275	3.40	0.0007
Cation Flux					
	Intercept	0.846	5.1	6.37	0.0013
-464.8108	TMI[1]	0.0578	1275	35.74	<0.001
	ONI[2]	-0.0673	1275	-8.8	<0.001
	TMI * ONI	0.0069	1275	3.41	0.0007
Sediment Flux					
	Intercept	1.294	8.4	28.11	<0.001
-114.2431	TMI[1]	0.0560	17.86	17.86	<0.001
	ONI[2]	0.1334	3.36	3.36	0.0009
	TMI * ONI	-0.0075	-1.94	-1.94	0.0532

[1]TMI = tri-monthly interval (e.g. DJF)

[2]ONI = oceanic niño index value

Table S10: Data Summary and r^2 values for weathering equations

Watershed	Daily hydrological data	Daily suspended sediment data	Individual Chemistry Measurements (yrs of record)	Total # of chemistry measurements	Na	K	Ca	Mg
			North side of canal					
Gatun	1998 - 2015	2005 - 2015	2003 -2005, 2007, 2011, 2013, 2014	100	0.90	0.96	0.84	0.93
Boqueron	1998 - 2015	2005 - 2015	2008 -2010, 2013, 2014	55	0.88	0.84	0.69	0.75
Pequini	1998 - 2015	2005 - 2015	2003 -2005, 2007 - 2011, 2013, 2014	112	0.92	0.88	0.78	0.91
Chagres	1998 - 2015	2005 - 2015	2003 -2005, 2007 -2011, 2013, 2014	99	0.57	0.80	0.57	0.84
Indio Este	2008 - 2015	N/A	2013 -2014	24	0.87	0.87	0.98	0.88
			South side of canal					
Ciri Grande	1998 - 2015	2005 - 2015	2003 -2005, 2007, 2011, 2013, 2014	78	0.68	0.89	0.74	0.77
Trinidad	1998 - 2015	2005 - 2015	2003 -2005, 2007, 2011, 2013, 2014	79	0.79	0.93	0.80	0.88
Cano Quebrado	2005 - 2015	2005 - 2015	2010	9	0.98	0.92	0.96	0.83

Table 11: ESA-CII landcover classes in study area

ESA-CII Class	Label
10	Cropland, rainfed
11	Herbaceous cover
12	Tree or shrub cover
20	Cropland, irrigated or post flooding
30	Mosaic cropland (>50%) / natural vegetation (tree, shrub, herbaceous cover) (<50%)
40	Mosaic natural vegetation (tree, shrub, herbaceous cover) (>50%) / cropland (<50%)
50	Tree cover, broadleaved, evergreen, closed to open (>15%)
60	Tree cover, broadleaved, deciduous, closed to open (>15%)
80	Tree cover, needleleaved, deciduous, closed to open (>15%)
100	Mosaic tree and shrub (>50%) / herbaceous cover (<50%)
110	Mosaic herbaceous cover (>50%) / tree and shrub (<50%)
120	Shrubland
130	Grassland
150	Sparse vegetation (tree, shrub, herbaceous cover) (<15%)
160	Tree cover, flooded, fresh or brakish water
170	Tree cover, flooded, saline water
180	Shrub or herbaceous cover, flooded, fresh/saline/brakish water
190	Urban areas
200	Bare areas
210	Water bodies

Table S12: Landcover reclassification

Class	Label	ESA-CII Class
1	Crops	10 & 20
2	Mosaic	12, 30, 40, 100 & 110
3	Trees	50, 60 & 80
4	Shrubs	120
5	Grass	11 & 130
6	Flooded	160, 170, & 180
7	Urban	190
8	Bare	150 & 200
9	Water	210
